# Phenotypic and Functional Heterogeneity of Monocytes and Macrophages

**DOI:** 10.3390/ijms241914525

**Published:** 2023-09-25

**Authors:** Lars Hellman

**Affiliations:** Department of Cell and Molecular Biology, Uppsala University, Uppsala, The Biomedical Center, Box 596, SE-751 24 Uppsala, Sweden; lars.hellman@icm.uu.se; Tel.: +46-(0)18-471-4532; Fax: +46-(0)18-471-4862

Macrophages are likely to be the first immune cells to have appeared during the evolution of multicellular organisms. Macrophages or macrophage-like cells with phagocytic activity have been found in cnidarians, one of the first groups of multicellular organisms to appear on Earth [1]. Macrophages are also present in essentially all human organs, and the macrophage populations of the different human organs often have different phenotypes and functions. All human macrophages were for many years thought to have originated from circulating monocytes, which after being recruited to the target organ were induced by cell-to-cell contacts and soluble mediators to become macrophages with features specific for that organ [2,3,4]. However, relatively recently, this view has been challenged by evidence that a majority of macrophage populations totally or almost totally originate from three waves of macrophage precursors during embryogenic development: first, from mesenchymal cells of the yolk sac wall; then, from the fetal liver; and finally, from the hemogenic endothelium of the aorta–gonad–mesonephral zone (Figure 1A) [3,5,6,7,8,9,10]. Many of these macrophage subpopulations seems to be maintained primarily by local proliferation, whereas others are repopulated by adult hematopoietic stem-cell-derived monocytes [2,4]. 

Macrophages of different organs often have at least partly different functions. Macrophages of the liver are called Kupffer cells. They are central players in the process of removing damaged proteins, apoptotic cells, and immune complexes from circulation. Macrophages in the lung, named alveolar macrophages, take part in the clearance of bacteria, viruses, and non-infectious particles that enter the lung during active breathing. There are also specific macrophages in the brain (named microglia cells), in the kidney, in the peritoneum, in the joints, in the spleen, in the intestinal region, and in many other places of the body. The specific proteins they express, their phenotype, and their functions differ between organs. By employing single-cell and quantitative transcriptomics, the differences in the phenotypes of these macrophage subpopulations are presently being analyzed, and a pattern of quite marked differences in phenotype is emerging (Figure 1) [11,12]. 

In one of the articles featured in this Special Issue of *IJMS*, a quantitative analysis of the transcriptome of mouse peritoneal macrophages has been performed [12]. In this article, the authors show that these macrophages, in addition to the classical macrophage proteins, such as lysozyme, high levels of lysosomal proteins, and several Fc receptors for IgG, also express high levels of several complement and coagulation components. These peritoneal macrophages express high levels of the three subunits of the first component of the classical pathway of complement activation, C1q; one of the components of the alternative complement activation cascade, factor P or properdin; one of the activators of the mannose pathway, ficolin; and also substantial amounts of C4a and factor H [12]. Of the coagulation components, it is primarily FV that is found in high amounts in these peritoneal macrophages [12]. In addition, FX, FVII, and complement factor B were expressed by these macrophages [12]. Interestingly, the liver, which has been considered the major producer of most complement and coagulation components, lacks the expression, or shows very low levels, of the majority of the proteins that were detected in high levels in these peritoneal macrophages. This finding indicates that tissue macrophages are major players in both of these systems via the local production of these complement and coagulation components [12]. How this interplay takes place has not yet been analyzed but is an interesting area of future research. High-resolution quantitative transcriptomics would also be highly interesting for studies of other macrophage populations, both mouse and human, to further dissect the specific role these subpopulations play in immunity. Single-cell studies have provided interesting insights, but a more quantitative analysis would be very informative for our understanding of the complex world of macrophage heterogeneity. The specific functions possessed by various macrophage populations are of major importance for our understanding of their general role in immunity and normal tissue homeostasis. For example, what specific role do lung macrophages play in the homeostasis of lung surfactants, and what role do microglia cells play in regulating the environment of brain cell functions?

If monocytes are not the major source of tissue macrophages, then what is their primary role in immunity? In the second article presented in this Special Issue, the authors show that circulating human monocytes are able to produce exceptionally high levels of mRNA for a highly selective set of inflammatory cytokines and chemokines [13]. Of the cytokines produced by these cells, we find that essentially only the classical inflammatory cytokines, IL-1α, IL-1β, IL-6, and TNF-α, increase in expression. The level of upregulation is also remarkably strong. IL-6 increases from 0.1 to 7500 reads, which is a 75,000-times increase after only 4 h of incubation in the presence of LPS [13]. One of the chemokines, IL-8, becomes the most highly expressed gene in the cell. IL-8 exceeds the former top transcript, lysozyme, by 50% [13]. These results show that monocytes are very sensitive sensors of infection that within minutes to hours can produce massive amounts of inflammatory cytokines and chemokines to initiate an inflammatory response. However, they have also been shown to be able to enter tissues during inflammation, and to differentiate into macrophage-like cells or to retain their monocyte characteristics within the tissue [4]. Therefore, they are highly sensitive circulating sensors of infection, but are also able to help other immune cells, including tissue-resident macrophages, in the affected tissue during infection. An important question here is what happens to these inflammatory monocyte-derived macrophages when the infection is cleared. This is not fully known, but may most likely involve the apoptosis of the majority of these cells. Experiments using the macrophage ablation of the liver have shown that the remaining resident yolk-sac-derived macrophages relatively rapidly repopulate the liver sinuses and only very few (2–5% of the total liver macrophages) are monocyte-derived, even after initial heavy depletion [4]. However, in other organs like the lung, the monocyte-derived macrophages can be long-lived and stay for extended periods, becoming almost indistinguishable in phenotype from their yolk-sac-derived counterparts [4]. 

Monocytes, like macrophages, can be separated into different subpopulations depending on which proteins they express and also different progenitor populations [14,15,16]. At least two distinct populations of circulating monocytes have been identified: those that express the low-affinity IgG receptor CD16, and those that do not (Figure 1B). In the third article presented in this Special Issue, the authors have performed a detailed analysis of the literature to obtain an overview of the abundance and relationship between these two populations [16]. The interplay between these two possible intermediates and other subpopulations seems very complex. The percentage of these two subpopulations within the total monocyte population has been found to be highly variable depending on inflammatory condition, type of analysis, and species, indicating a complex pattern of regulation within and between these two populations (Figure 1B) [16]. This article clearly shows the complexity of this field, and gives a detailed review of the literature [16]. 

There are major obstacles involved in studying human monocyte and macrophage biology compared to other species, where it is often possible to use knock out and transgene technologies. Several model systems for studies of human monocyte/macrophage biology have therefore been developed. One such model system is humanized mice, where human cells or one or several genes for human proteins are introduced into the mouse genome to facilitate the analysis of a particular human immune cell or protein, regarding its role in immunity [17,18,19]. Although these models represent an important step, these systems are artificial as cells generally rely on a large number of cellular contacts involving a large number of protein–protein interactions, protein–carbohydrate, or protein–lipid interactions, in addition to an array of different growth and differentiation factors. Another model system for studies of human immune cell biology is cell lines. In such cells, one can look at the role of individual proteins in cell signaling, vesicle transport, post-translational processing, and many other processes of importance in the role of the corresponding cell in vivo. However, for many of these questions, it is important to know the phenotype of the cell and how similar the cell is to its in vivo counterpart. In the fourth article featured in this Special Issue, the authors analyze the phenotype of two commonly used monocytic cell lines, MonoMac-6 and THP-1 [20]. Both of these cell lines have been extensively used in studies of monocyte biology. A screening of PubMed for different articles on the use of these two cell lines showed 351 hits for MonoMac-6 and more than 14,500 for THP-1, indicating their extensive use as a model system for various studies of human monocyte biology [20]. Quantitative transcriptomics showed that both of these cell lines have no or very little resemblance to circulating human monocytes. The transcriptome instead showed that they represent an early stage in neutrophil development and express very few monocyte-related immune proteins (Figure 1C) [20]. This further stresses the difficulties in finding good model systems for studies of human monocyte–macrophage biology.

In the fifth article presented in this Special Issue, the authors review the current state of knowledge regarding the biology of the Kupffer cells of the liver, the largest population of resident tissue macrophages in the body [4]. Estimates indicate that 80–90% of all the macrophages in different mammals are found in the liver [21]. These liver macrophages are very important for the clearance of circulating immune complexes, as well as the clearance of apoptotic cells and damaged blood proteins, in addition to sensing infection and removing potentially incoming pathogens, primarily bacteria, through the portal vein [22,23,24]. High heterogeneity has been observed within the liver macrophage population [4,25]. The absolute majority, more than 90%, seem to be derived from monocytes of the fetal liver (Figure 1A) [4]. These initially yolk-sac-derived macrophages seem to be very stable populations, as mentioned above, and even after heavy experimental depletion they repopulate the liver, leaving little room for bone-marrow-derived monocytes to enter the tissue [4]. Interestingly, these liver macrophages also seem to respond very differently to LPS compared to circulating monocytes, as discussed above. In these Kupffer cells, LPS may instead induce an anti-inflammatory phenotype by suppressing the activation of helper T cells and attracting regulatory T cells [4]. 

In summary, this Special Issue presents key findings in both macrophage and monocyte biology regarding the role of tissue macrophages in the regulation of the complement and coagulation systems, the potent role of circulating human monocytes in the induction of an inflammatory response, and the difficulty of finding good model systems to study human monocyte–macrophage biology. It has also resulted in a detailed summary of published material concerning the complex pattern of monocyte subpopulations and the biology of one of the key macrophage populations of the human body, the Kupffer cells of the liver. These latter reviews serve as a good basis for future studies of these subpopulations in human monocyte and macrophage biology and medicine.

## Figures and Tables

**Figure 1:**
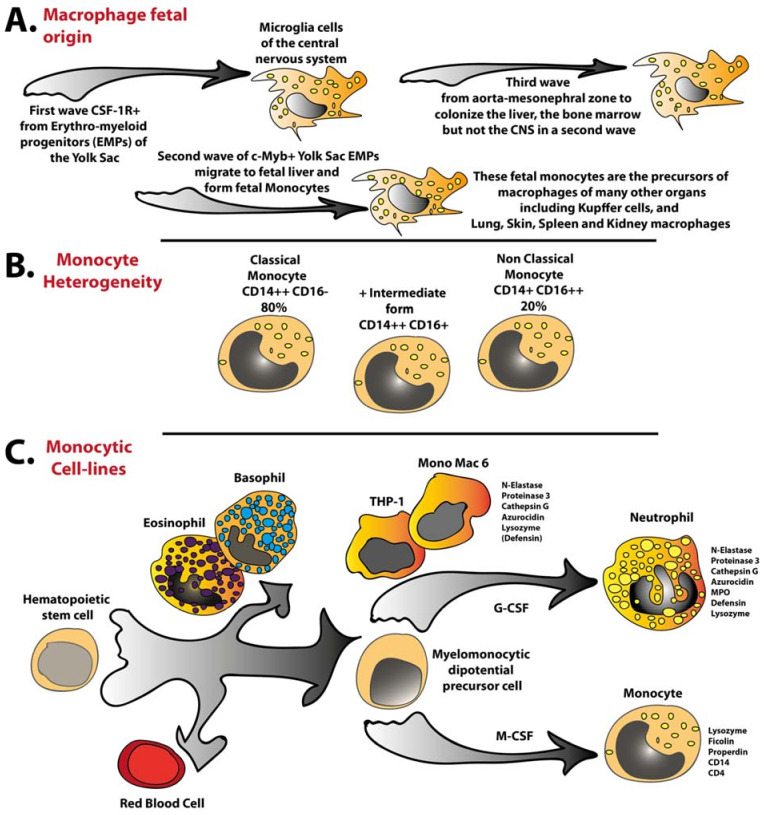
**Schematic representations of three important areas of mammalian monocyte and macrophage biology.** Panel (**A**) shows the three waves of embryonic macrophage migrations during the embryonic development of the developing body. Panel (**B**) shows a schematic representation of the two main human monocyte populations, the classical CD14-positive and CD16-negative cells that represent the majority of circulating monocytes, and the minor non-classical monocytes that are CD16-positive. The non-classical monocytes are thought to originate from the classical by moving through an intermediate stage that shows high CD14 levels and low levels of CD16. Panel (**C**) shows the phenotype of two cell lines, MonoMac6 and THP-1, that are frequently used as model systems for in vitro studies of human monocyte biology.
